# Alteration of humoral, cellular and cytokine immune response to inactivated influenza vaccine in patients with Sickle Cell Disease

**DOI:** 10.1371/journal.pone.0223991

**Published:** 2019-10-10

**Authors:** Carole Nagant, Cyril Barbezange, Laurence Dedeken, Tatiana Besse-Hammer, Isabelle Thomas, Bhavna Mahadeb, André Efira, Alice Ferster, Francis Corazza

**Affiliations:** 1 Immunology Department, LHUB-ULB, Université libre de Bruxelles, Brussels, Belgium; 2 National Influenza Centre, Sciensano, Brussels, Belgium; 3 Department of Hematology Oncology, Hôpital Universitaire des Enfants Reine Fabiola, Université libre de Bruxelles, Brussels, Belgium; 4 Department of Hematology Oncology, Centre Hospitalier Universitaire Brugmann, Brussels, Belgium; 5 Microbiology Department, LHUB-ULB, Université libre de Bruxelles, Brussels, Belgium; Icahn School of Medicine at Mount Sinai, UNITED STATES

## Abstract

**Introduction:**

Patients suffering from Sickle Cell Disease (SCD) are at increased risk for complications due to influenza virus. Annual influenza vaccination is strongly recommended but few clinical studies have assessed its immunogenicity in individuals with SCD. The aim of this study was to explore the biological efficacy of annual influenza vaccination in SCD patients by characterizing both their humoral and cell-mediated immunity against influenza antigen. We also aimed to investigate these immunological responses among SCD individuals according to their treatment (hydroxyurea (HU), chronic blood transfusions (CT), both HU and CT or none of them).

**Methods:**

Seventy-two SCD patients (49 receiving HU, 9 on CT, 7 with both and 7 without treatment) and 30 healthy controls were included in the study. All subjects received the tetravalent influenza α-RIX-Tetra® vaccine from the 2016–2017 or 2017–2018 season.

**Results:**

Protective anti-influenza HAI titers were obtained for the majority of SCD patients one month after vaccination but seroconversion rates in patient groups were strongly decreased compared to controls. Immune cell counts, particularly cellular memory including memory T and memory B cells, were greatly reduced in SCD individuals. Functional activation assays confirmed a poorer CD8^+^ T cell memory. We also document an imbalance of cytokines after influenza vaccination in SCD individuals with an INFγ/IL-10 ratio (Th1-type/Treg-type response) significantly lower in the SCD cohort.

**Conclusion:**

SCD patients undergoing CT showed altered immune regulation as compared to other treatment subgroups. Altogether, the cytokine imbalance, the high regulatory T cell levels and the low memory lymphocyte subset levels observed in the SCD cohort, namely for those on CT, suggest a poor ability of SCD patients to fight against influenza infection. Nevertheless, our serological data support current clinical practice for annual influenza vaccination, though immunogenicity to other vaccines involving immunological memory might be hampered in SCD patients and should be further investigated.

## Introduction

Sickle cell disease (SCD), one of the most common autosomal recessive disorders worldwide, is associated with numerous acute and chronic complications requiring immediate support. Actual strongly recommended therapies include chronic blood transfusions (CT) and hydroxyurea (HU) [1]. While there is strong evidence to support the use of HU in patients with recurrent vaso-occlusive crises, acute chest syndrome or chronic anaemia, regular red blood cell (RBC) transfusion to maintain HbS < 30% is recommended for patients being at high risk for stroke. Furthermore, chronic RBC transfusions prevent recurrent ischemic stroke and decrease the incidence of acute chest syndrome (ACS) [2–4].

Despite common use of these therapies in SCD, little work has been done to investigate the impact of CT or HU therapy on immune functions in SCD patients. Immunological abnormalities and alterations in specific immune cell subsets have been reported in individuals with SCD undergoing CT [5–6].

SCD patients are also at increased risk for infections associated with greater morbidity and mortality [7–8]. Influenza virus is a common cause of infection in patients with SCD, while complications from influenza infection are often more severe and more frequently fatal in SCD individuals [9]. The number of influenza-related hospitalizations is up to 56 times higher in SCD than in the general pediatric population [10–11]. Influenza infection is thus a clinically important problem for SCD individuals and annual influenza vaccination is therefore strongly recommended starting at 6 months of age [12]. However, the efficacy of this vaccination strategy has been poorly investigated in SCD cohorts. Antibody titers following pneumococcal vaccination in SCD individuals have been shown to decline over a year suggesting the need for re-vaccination [13]. Moreover, the impact of HU or CT, which act as potential immune modulators, on influenza vaccination is unclear. Purohit et al. [14] showed decreased seroprotection following inactivated influenza A(H1N1)pdm09 vaccine in patient with SCD receiving CT, but, few other clinical studies have reported influenza vaccine immunogenicity in individuals with SCD.

The objective of this study was therefore to explore the biological efficacy of annual influenza vaccination in SCD patients by characterizing both their humoral and cell-mediated immunity against influenza antigen. We also aimed to investigate these immunological responses among SCD individuals according to their treatment (HU, CT, both HU and CT or none of them).

## Methods

### Population description and study design

Patients with SCD and healthy controls were enrolled in the study spanning two influenza seasons, between 2016 and 2018. The recruitment of SCD patients was done by the Hematology-Oncology Departments of the Hôpital Universitaire des Enfants Reine Fabiola (HUDERF-ULB) and Centre Hospitalier Universitaire de Brugmann (CHU Brugmann). Exclusion criteria included a poor peripheral venous access, transplanted patients, splenectomy and patients under the age of 3 years as they received a different influenza vaccinal schedule.

Patients were classified as being on CT therapy if they had recently received >3 consecutive RBC transfusions. All patients on CT enrolled in this study were on exchange transfusion therapy (manual or automated).

The control group consisted of voluntarily vaccinated medical staff.

All subjects benefited from the α-RIX-Tetra® vaccine (102 subjects for the 2016–2017 season and 6 subjects for the 2017–2018 season). The H1N1 component for the 2016–2017 season was A/California/07/2009 and for the 2017–2018 season A/Michigan/45/2015.

Blood samples were collected on the day of vaccination (T0) and 1 (T1), 3 (T3) and 6 (T6) months after administration of the influenza vaccine.

All participants included in the study provided clear written and suitably informed consent for study participation, in compliance with the principles of the Helsinki Declaration [15]. For minor participants under the age of 18, consent was obtained from parents. The study was approved by the Ethics Committee of Reine Fabiola Children's Hospital and the Brugmann Hospital (CE2016/114).

### Hemagglutination inhibition assay

Anti-influenza antibodies were measured by hemagglutination inhibition (HAI) against A/California/07/2009 (H1N1pdm09) strain component of the vaccine. For each subject, HAI titer was established at day 0 and 1, 3 and 6 months after vaccination. The seroconversion rate (at least 4-fold increase in HAI titers) at T1, T3, T6 was calculated with respect to the titer at T0. HAI titers of 1:40 or more were considered positive for seroprotection [16].

### Immunophenotype analysis

Lymphocyte populations and subsets were evaluated on whole blood via multiparametric flow cytometry (MFC). The following lymphocyte populations were identified: CD3^+^ T cells, CD19^+^ B cells, CD16^+^56^+^ NK cells, CD4^+^ T cells (CD4^+^/CD8^−^ T cells), CD8^+^ T cells (CD8^+^/CD4^−^T cells) and T regulatory (Treg) cells (CD4^+^/CD25^high^/CD127^low^/FoxP3^+^). In addition, among T cells, the following sub-populations were defined: naive T cells (Tn) characterized as CD45RO^-^/CD27^+^; central memory (CM) as CD45RO^+^/CD27^+^; effector memory (EM) as CD45RO^+^/CD27^−^ and terminally-differentiated effector cells (TD) as CD45RO^-^/CD27^−^ for both the CD4^+^ and CD8^+^ T cell populations. Memory B cells were identified as CD19^+^CD27^+^. Flow cytometry data was acquired on BD FACSCanto^™^ II cytometer and analysed using BD FACS Diva software (BD Biosciences, San Jose, CA, USA). The absolute counts for lymphocyte subsets were obtained by the following formula: Absolute count = subset (%) x CD19^+^/CD4^+^/CD8^+^ (%lymphocyte) x total lymphocyte number (10^3^/μL).

### Lymphocyte activation test

A lymphocyte activation test was performed as previously described [17]. Fresh blood samples were cultured for 24h in either standard cell culture medium (basal condition), culture medium supplemented with phytohemagglutinin (PHA) 10 μg/ml (maximal activation) or culture medium supplemented with influenza vaccine antigens 3 μg/mL (α-RIX-Tetra® vaccine 2016–2017). The expression of the early activation marker CD69^+^ [18] was measured on CD4^+^ and CD8^+^ T cells using MFC BD FACSCanto^™^ II. The absence of CD69^+^ expression (< 1% lymphocytes) after incubation in basal condition was classified as negative control. Results were normalised by calculating a ratio of CD69^+^ expression after influenza vaccine stimulation *versus* in basal conditions. ((%CD69^+^ after influenza vaccine stimulation) / (%CD69^+^ in basal conditions)).

### Cytokine assays

Whole blood was incubated with culture medium supplemented with either influenza vaccine antigen 3 μg/mL (α-RIX-Tetra® vaccine 2016–2017, stimulated condition), PHA 10 μg/ml (maximal activation) or plain culture medium (basal condition) for 72h. Multiplex cytokine measurements (IL-10, IL-4, IL-6, IFNγ and TGFβ1) on cell culture supernatans were performed using a plate-based electrochemiluminescence assay according to manufacturer’s instructions (MSD, Meso Scale Discovery, Rockville, MD, USA).

### Heme-oxygenase 1 (HO-1) expression in whole cell lysate

Whole blood samples were collected and red blood cells were lysed by incubating with NH_4_Cl at 37°C for 2 minutes. After several washing steps, the cell pellet was suspended in phosphate buffered saline (PBS). Samples were conserved at -80°C until the day of the experiment. HO-1 concentration in the cell lysate was assessed using an ELISA-based enzyme activity assay according to the manufacturer’s recommendations (HO-1 ELISA Kit, EnzoLifeSciences). Staining was measured with a 450 nm microplate reader (DS2, Dynex, USA). HO-1 concentrations were calculated using a standard curve generated with a calibrated HO-1 protein standard.

### Statistical analysis

Statistical analysis of data was performed using GraphPad Prism 5 (GraphPad Software, San Diego, CA) (*p<0.05; **p<0.01; ***p<0.005; ns, nonsignificant). Nonparametric Mann-Whitney U test was used for the comparison of two groups and nonparametric Kruskal-Wallis for the analysis of variance, followed by a Dunn’s multiple comparison test was performed for the comparison of more than two groups. Fisher’s exact test was used to compare the rates of seroconversion.

We performed a multivariable analysis by principal component analysis (PCA) in order to corroborate the distinct immunological profile of a subgroups of patients (SPSS, version 25).

## Results

### Patient characteristics

Seventy-two (72) patients with SCD and 30 healthy controls were included in the study. The median age was 16 years (range: 3–57) for the SCD cohort and 51 years (range: 24–78) for the healthy cohort. Unfortunately, it was ethically difficult to include younger healthy controls as it is not recommended to vaccinate them against the flu (Conseil Supérieur de la Santé. Guide de vaccination, Bruxelles, 2009—n° 8586). The distribution of SCD patients according to their therapeutic regimen is shown in Table 1; 49 patients were treated with HU, 9 patients undergoing CT, 7 SCD patients were treated with both HU and CT and 7 patients had no disease-modifying therapy (no DMT). Most patients are treated with HU alone explaining the smaller size of the CT subgroup.

**Table 1:** Distribution of the subjects participating to the study.

			SUB-GROUPS OF SCD PATIENTS			
	Healthy control	SCD PATIENTS	HU^a^	CT^b^	CT+HU	No DMT^c^
**Age**^**d**^	51(24–78)	16(3–57)	15(3–57)	16(8–40)	12(6–19)	18(6–35)
**F/M**	19/11	41/31	33/16	1/8	2/5	5/2
**n T0**	30	72	49	9	7	7
**n T1**	28	68	45	9	7	7
**n T3**	30	69	46	9	7	7
**n T6**	27	56	39	8	7	2

^a^ Hydroxyurea (HU)

^b^ Chronic transfusion (CT)

^c^ No Disease modifying therapy (DMT)

^d^ Median age in years (range)

### Differences in HAI titers between SCD patients and healthy donors

Serum antibody titers were measured by a hemagglutination inhibition assay.

**Fig 1:**
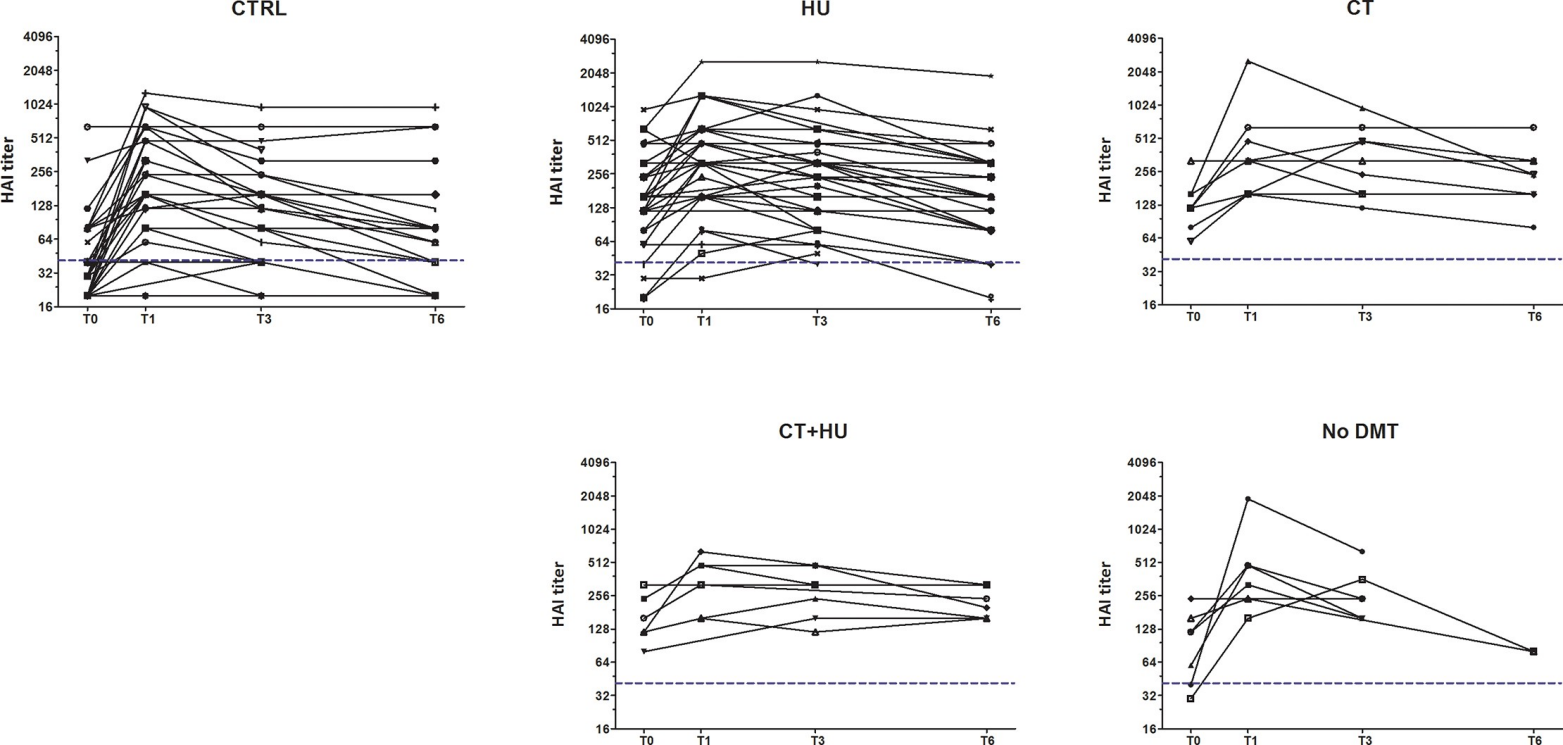
Kinetics of influenza HAI titers. Evolution of HAI titers measured at 0 (T0), 1 (T1), 3 (T3) and 6 (T6) months after vaccination for each subject. **(A)** Control group **(B)** SCD patients divided by treatment group. HU, hydroxyurea; CT, chronic transfusion; CT+HU both chronic transfusion and hydroxyurea; No DMT, No Disease modifying therapy. The dotted lines represent protective titer (1:40).

Before vaccination, the median anti-H1N1 HAI titer was significantly higher for SCD patients compared to healthy subjects (control, 1:30(1:20–1:80); SCD, 1:160(1:120–1:240); p <0.0001). Titers remained significantly higher for the SCD group at T3 and T6. Among the SCD subgroups, no significant difference in anti-influenza HAI titers was observed with respect to treatment (Fig 1).

One month after vaccination, 68% (19/28) of the control group seroconverted compared to 22% (15/67) of the combined SCD group (all treatments) (Fisher's test, T1, p < 0.0001***) (Table 2). A significantly lower seroconversion rate was also observed in the SCD group compared to the healthy control at T3 and T6 (T3, p = 0.0098**; T6, p = 0.0136*). Significant difference of seroconvertion rate at T1 was maintained when comparing groups related to age, to avoid age bias (p < 0.0001***). Within the group of SCD patients, no significant difference in seroconversion was observed related to treatment.

Neither the control group (p NS) nor the SCD group (p NS) showed significant decrease in seroconversion between T1 and T3. In contrast, seroconversion was significantly decreased at T6 for both groups (control, p = 0.0018*; SCD, p = 0.0104*).

**Table 2:** Seroconversion and seroprotection rate.

	SEROCONVERSION						SEROPROTECTION							
	T1		T3		T6		T0		T1		T3		T6	
	%	(n/n total)	%	(n/n total)	%	(n/n total)	%	(n/n total)	%	(n/n total)	%	(n/n total)	%	(n/n total)
**HU**^**a**^	22	(10/45)	11	(5/45)	3	(1/38)	92	(44/48)	92	(44/48)	100	(45/45)	97	(36/37)
**CT**^**b**^	33	(3/9)	33	(3/9)	25	(2/8)	100	(9/9)	100	(9/9)	100	(9/9)	100	(8/8)
**CT+HU**	17	(1/6)	17	(1/6)	0	(0/6)	100	(7/7)	100	(7/7)	100	(7/7)	100	(7/7)
**No DMT**^**c**^	14	(1/7)	33	(2/6)	0	(0/2)	86	(6/7)	100	(8/8)	100	(6/6)	100	(2/2)
**SCD**	22	(15/67)*	17	(11/66)*	6	(3/54)*	93	(66/71)	94	(68/72)	100	(67/67)	98	(53/54)
**SCD>16y**	30	(11/36)**	29	(8/28)**	12	(3/26)**	92	(34/37)	97	(37/38)	100	(37/37)	97	(28/29)
**CTRL**	68	(19/28)	43	(13/30)	26	(7/27)	47	(14/30)	96	(27/28)	93	(28/30)	78	(21/27)
**P-value**		0.0051*		0.0667*		0.1710*								
**P-value**		<0.0001**		0.0098**		0.0136**								

A 4-fold increase in HAI titers between two paired samples is considered as positive for seroconversion. Seroconversion at T1, T3, T6 was defined relative to the titer at T0. Fisher exact test was used to compare the seroconversion rate between controls and SCD patients. HAI titers of 1:40 or more are considered positive for seroprotection.

*SCD vs CTRL

**SCD>16y vs CTRL

^a^ Hydroxyurea (HU)

^b^ Chronic transfusion (CT)

^c^ No Disease modifying therapy (DMT)

Before vaccination (T0), 60% (18/30) of healthy subjects already had protective levels and 93% (66/71) of individuals with SCD. Among healthy subjects, we compared seroconversion rate at T1, T3 and T6 according to their previous vaccination status. There was no statistically significant difference between those previously vaccinated (HV, history of vaccination) versus those previously unvaccinated (No HV, No history of vaccination): T1 (HV 4(1,8–12); No HV 8(6–8); p NS); T3 (HV 2(1,5–4,65); No HV 4(1,5–8); p NS); T6 (HV 2(1,0–3,67); No HV 1,65(0,95–4); p NS). Protective anti-influenza HAI levels were detected for the majority of the subjects up to T6 (Table 2).

### Alterations in immune cell counts in patients with SCD

**Fig 2:**
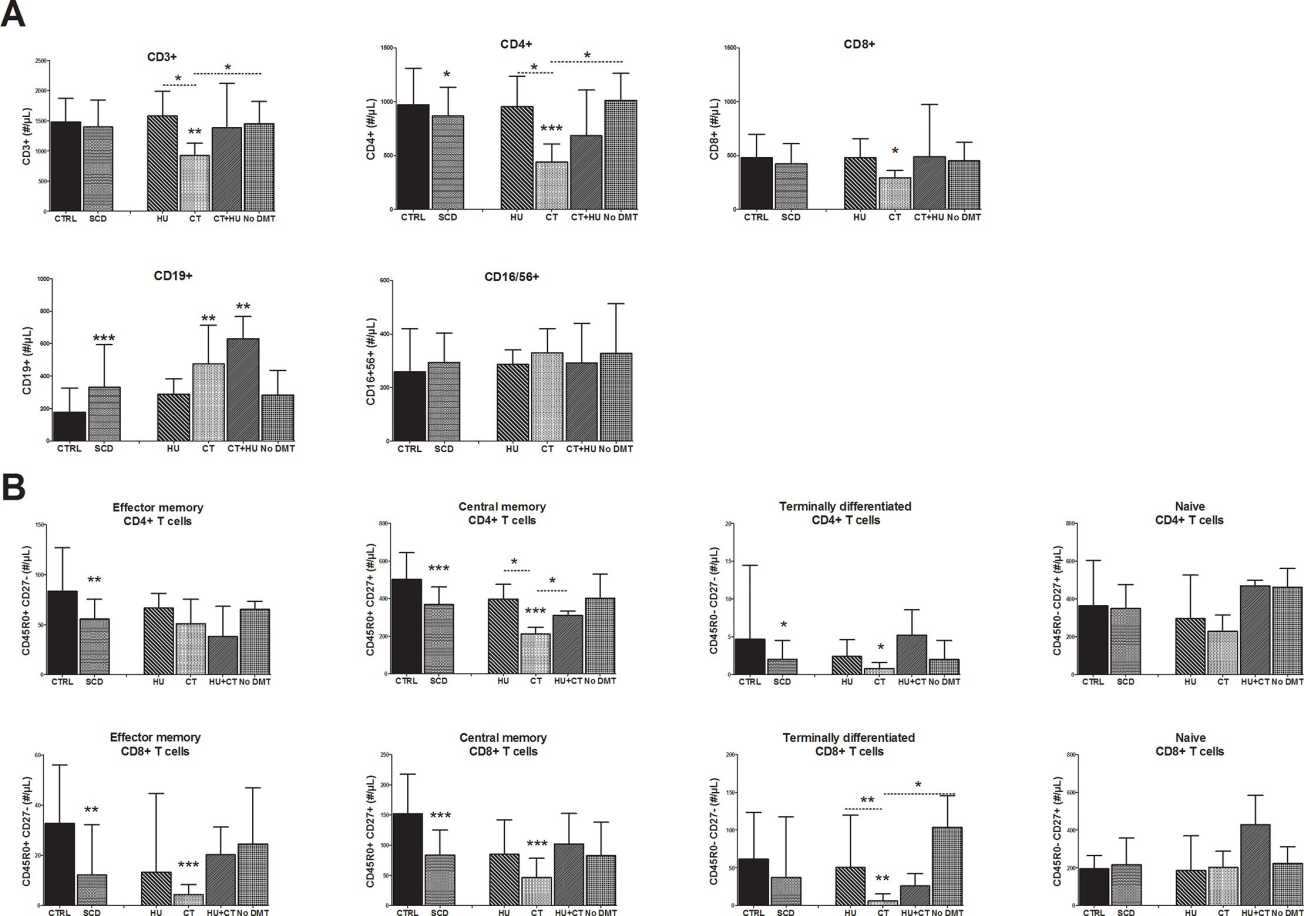
Comparison of lymphocyte cell count in SCD patient treatment groups *versus* healthy controls. **(A)** Total CD3^+^, CD4^+^, CD8^+^, CD19^+^, CD16/56^+^ cell counts. **(B)** CD4^+^ T cell subpopulations and CD8^+^ T cell subpopulations cell counts. Comparison between SCD combined groups and healthy control groups were assessed by two tailed nonparametric Mann-Whitney test. Comparison between SCD subgroups according to their treatment and the healthy control group were assessed by the nonparametric Kruskal-Wallis analysis of variance, followed by a Dunn’s multiple comparison test. P-value above bar represent comparison to the healthy control group. Other significant associations are shown. Results of lymphocyte populations are represented as median ± interquartile range (IQR). Results are represented for individuals ≥ 16 years. HU, Hydroxyurea; CT, Chronic transfusion; No DMT, No Disease modifying therapy. *p<0.05; **p<0.01; ***p<0.005.

Absolute counts of lymphocyte populations (CD3^+^ T cells, CD4^+^ T cells, CD8^+^ T cells, CD19^+^ B cells, CD16^+^56^+^ NK cells) were evaluated by MFC for SCD patients and for healthy donors. Statistical analysis was performed only for subjects ≥ 16 years to avoid age bias for age-dependent parameters. Significant differences were observed for CD4^+^ T cells in the control group *versus* the combined SCD group. Moreover, SCD patients on CT had significantly lower CD3^+^ (p<0.01), CD4^+^ (p<0.005) and CD8^+^ (p<0.05) T cells compared to the control group and even compared to the SCD group receiving HU (p<0.05) (Fig 2). Conversely, SCD patients showed significantly higher absolute counts of B cells compared to the control group.

We further investigated T cell subsets among our populations. When analysed in a whole group, SCD patients had significantly lower CD4^+^ and CD8^+^ T memory cells compared to controls. This trend was observed for both EM and CM subsets (CD4^+^ EM p<0.01; CD4^+^ CM p<0.005; CD8^+^ EM p<0.005; CD8^+^ CM p<0.005) (Fig 2B). These low levels were still statistically significant for SCD patients on CT after an analysis of variance for CD4^+^ CM (p<0.005), CD8^+^ EM (p<0.005) and CD8^+^ CM (p<0.01) T cells. CD4^+^ (p<0.05) and CD8^+^ (p<0.01) TD effector cells were also significantly decreased in the SCD group on CT.

Same evolution of cell counts according to age (5–10 years and 10–16 years) was described for SCD patients and healthy paediatric population although levels were different [19].

Percentages and absolute values of total memory B cells were interpreted in relation to age-specific reference values [20]. SCD patients had lower total memory B cells compared to healthy controls at all ages (Table 3). As defined by the Paris classification of Piqueras et al. [21], the threshold value of 11% of total memory B cells for adults and children from 2 years of age was not reached in 61% (42/69) of SCD patients compared to 4% (1/27) of healthy subjects (Fisher's test, p <0.0001***).

**Table 3:** Total memory B cells according to age.

	*CD19*^*+*^*CD27*^*+*^* total memory B cells (/*μ*L)*					*CD19*^*+*^*CD27*^*+*^* total memory B cells (%)*				
	(/μL)	5–10y	10–15y	15–18y	>18y	(%)	5–10y	10–15y	15–18y	>18y
**Healthy control**	**Median**	241^(^*^)^	274^(^*^)^	241^(^*^)^	57	**Median**	23^(^*^)^	19^(^*^)^	21^(^*^)^	28
	**P10-P90**	131-406^(^*^)^	99-310^(^*^)^	64-376^(^*^)^	21–107	**P10-P90**	15-31^(^*^)^	12-29^(^*^)^	13-39^(^*^)^	16–54
**SCD patients**	**Median**	85	29	50	35	**Median**	10	5	9	11
	**P10-P90**	23–156	15–86	22–21	13–83	**P10-P90**	7–20	3–18	3–20	5–57
	**P-value**				0.007	**P-value**				<0.0001

P10 = 10^th^ percentile, P90 = 90^th^ percentile, y = year

^(*)^ Smet et al. 2011 [19]

### Lower lymphocyte activation in patients with SCD

**Fig 3:**
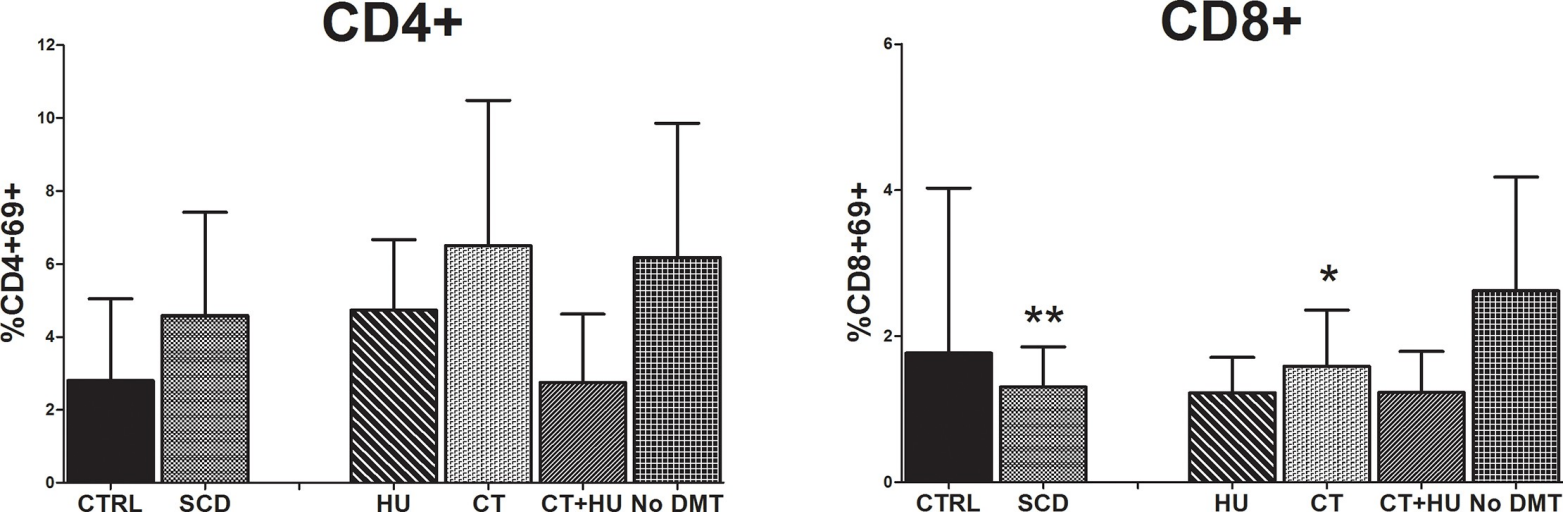
Lymphocyte transformation test (LTT) after activation with influenza vaccine. Results are expressed as the ratio of **(A)** (%CD4^+^CD69^+^ after influenza vaccine stimulation) / (%CD4^+^CD69^+^ in basal conditions) and **(B)** (%CD8^+^CD69^+^ after influenza vaccine stimulation) / (%CD8^+^CD69^+^ in basal conditions). Median ratio with IQR are represented for each group. HU, Hydroxyurea; CT, Chronic transfusion; No DMT, No Disease modifying therapy. *p<0.05; **p<0.01; ***p<0.005.

A functional test to assess sensitization of T cells to vaccine antigens in order to estimate the effectiveness of memory T cells was performed by evaluating the expression of an activation surface marker (CD69) on CD4^+^ and CD8^+^ T cells following 24h incubation with the influenza vaccine antigens. No significant difference was shown in the activation of CD4^+^ cells among the combined SCD group and the control group (control 2.8%(2.3–5.0); SCD 4.6%(2.3–7.4); p NS). However, SCD patients presented significantly lower activation of CD8^+^ cells after stimulation by the influenza vaccine antigens than the control group (control 1.8%(1.4–4.0); SCD 1.3%(0.9–1.9), p = 0.0073) (Fig 3) while responses to PHA (maximal stimulation) were vigorous and similar among all groups (CD4^+^CD69^+^, control 53,4%(33,5–63,1); SCD 58,0%(48,9–68,1) and CD8^+^CD69^+^, control 63,3%(56,2–71,3); SCD 72,3%(62,8–87,2)).

### Increased levels of cytokines and decreased INFγ/IL-10 ratio for SCD patients

**Fig 4:**
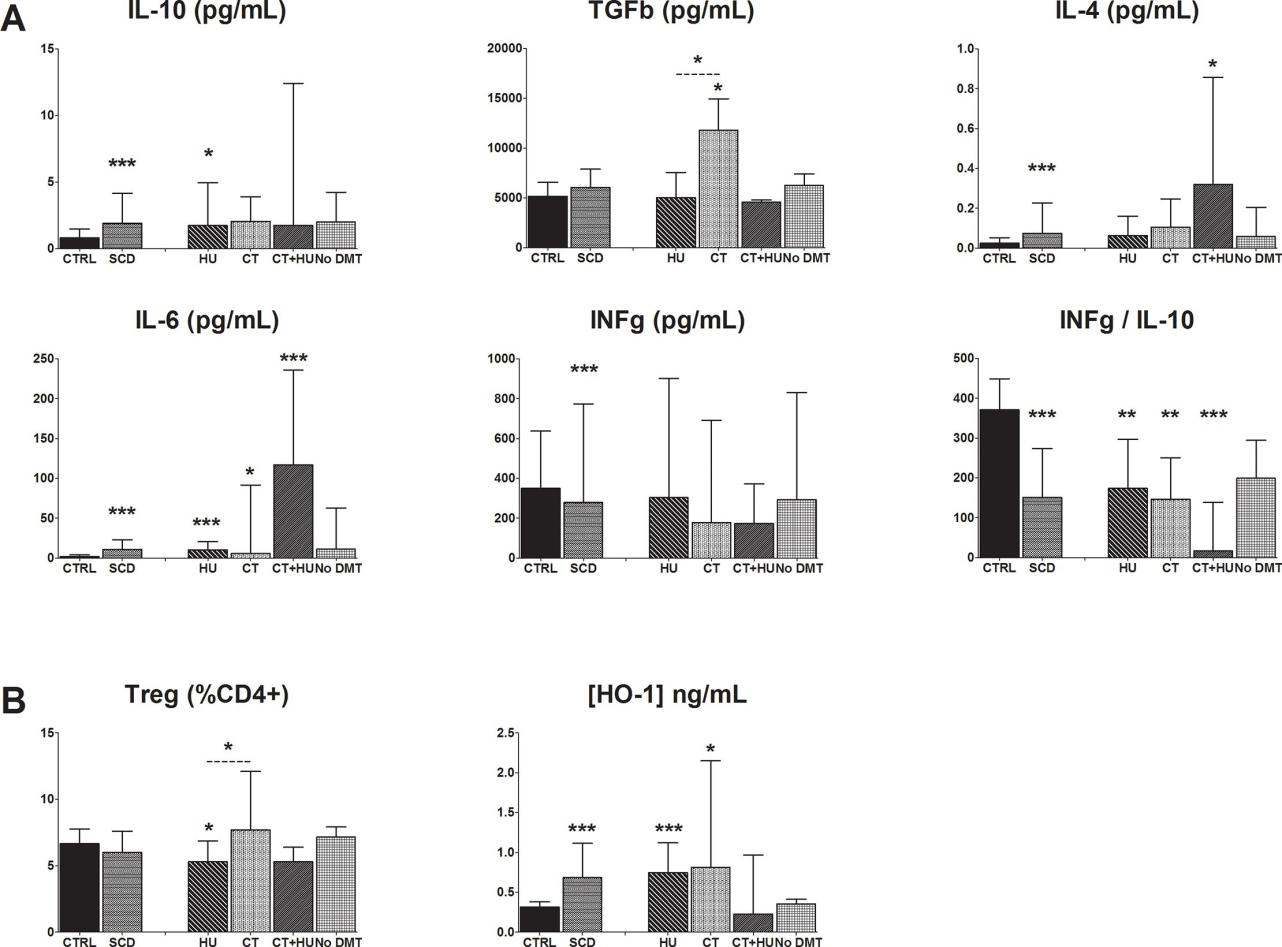
Distribution of cytokine levels, regulatory T cells and HO-1 concentration in SCD patient treatment groups *versus* healthy controls. **(A)** Levels of IL-10, TGFβ, IL-4, IL-6, INFγ and INFγ/IL-10 were assessed by a multiplex assay. **(B)** Levels of Treg cells were assessed by MFC and HO-1 concentration in cell lysate was evaluated by an ELISA assay. Comparison between SCD combined groups and healthy control groups were assessed by two tailed nonparametric Mann-Whitney test. Comparison between SCD subgroups according to their treatment and the healthy control group were assessed by the nonparametric Kruskal-Wallis analysis of variance, followed by a Dunn’s multiple comparison test. P-value above bar represent comparison to the healthy control group. Other significant associations are shown. HU, Hydroxyurea; CT, Chronic transfusion; No DMT, No Disease modifying therapy. *p<0.05; **p<0.01; ***p<0.005.

Anti- and pro-inflammatory cytokines were evaluated in culture medium after stimulation with influenza vaccine antigens. Significantly higher levels of IL-4, IL-6 and INFγ were observed in the combined SCD group when compared to the healthy donors group (p<0.005) (Fig 4A). SCD patients also had increased levels of the anti-inflammatory cytokines IL-10 (SCD on HU, p<0.05) and TGFβ (SCD on CT, p<0.05) (Fig 4A). Furthemore, higher proportions of Treg cells were observed in the SCD group under CT compared to SCD patients on HU (p<0.05) (Fig 4B).

The pro-/ anti-inflammatory cytokine ratio (INFγ/IL-10) was significantly lower in the SCD combined group compared to the control group (p<0.005). These lower levels remained significant for the SCD patients on HU, CT and receiving both treatments.

### Higher levels of heme-oxygenase 1 (HO-1) for SCD patients under treatment

HO-1 concentration was significantly higher in the SCD group compared to controls (control, 0.3 ng/mL (0.2–0.4); SCD, 0.7 ng/mL (0.3–1.1); p<0.0001). This difference remained significant for SCD patients under HU and CT (HU, 0.7 ng/mL (0.3–1.1); CT, 1.0 ng/mL (0.5–5.4); p<0.05) (Fig 4B).

### Principal component analysis

**Fig 5:**
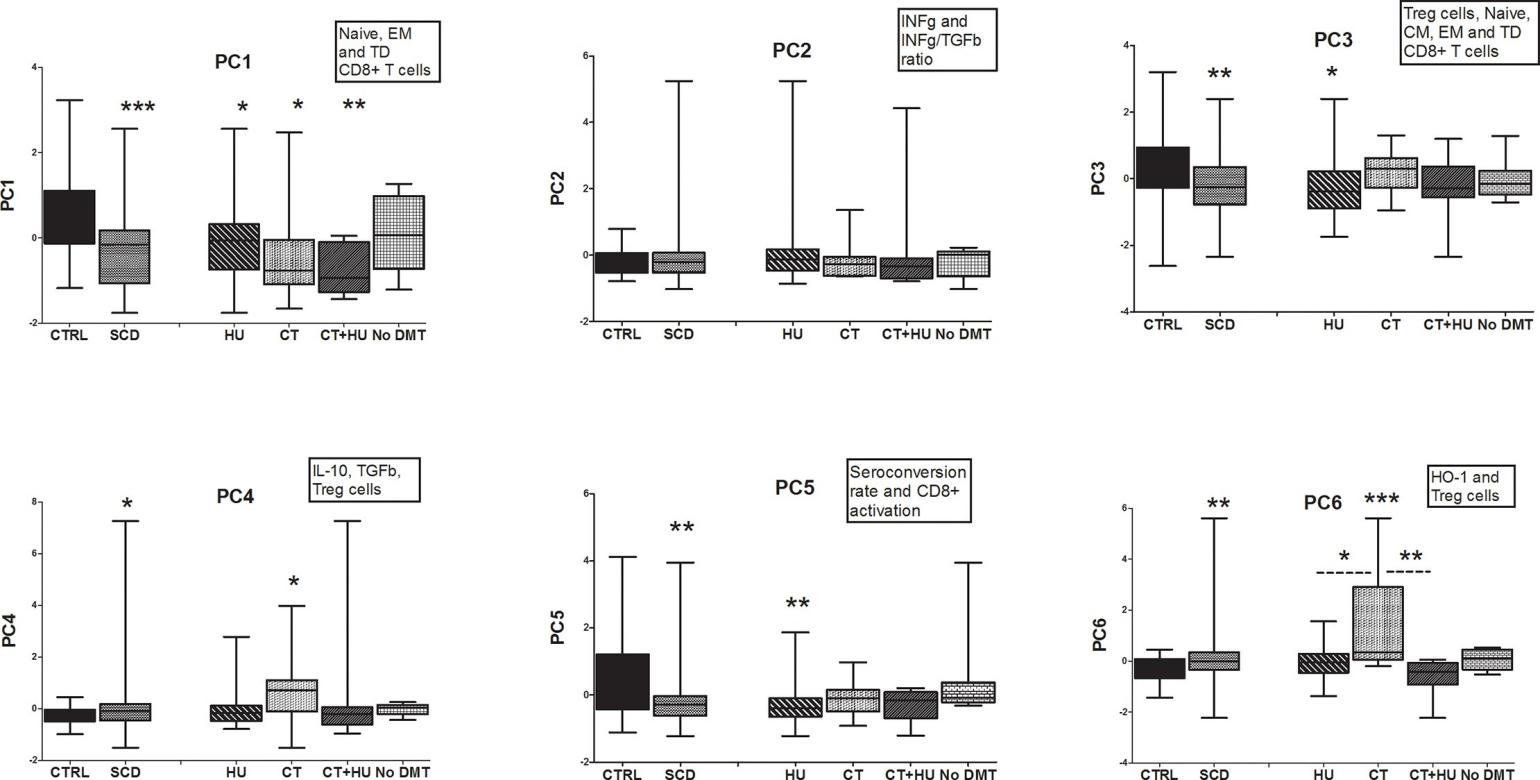
Principal component analysis. Box plots of PC1 to PC6 for SCD treatment groups compared to healthy controls. Comparison between groups was assessed by Kruskal-Wallis analysis of variance, followed by a Dunn’s multiple. The nonparametric Mann-Whitney U test was used for the comparison of SCD patients and healthy controls.

To corroborate the distinct immunological profile of a subgroups of patients we performed a multivariate analysis by PCA with the most relevant 12 parameters. Principal components (PC) were mainly composed of, for PC1: Naive, EM and TD CD8^+^ T cells; PC2: INFγ and INFγ/TGFβ ratio; PC3: Treg cells, Naive, CM, EM and TD CD8^+^ T cells; PC4: IL-10, TGFβ, Treg cells; PC5: seroconversion rate and CD8+ activation; PC6: HO-1 and Treg cells. PC1 accounted for 21% of the variance of the data; PC2 for 16%; PC3 for 12%; PC4 for 10%; PC5 for 9% and PC6 for 9% of the variance. Cumulative variance for the 6 PC was of 76%. PC1, PC3-6 were significantly different between controls and SCD patients. The group of SCD patients on CT was distinct from the others for the PC1, PC4 and PC6 analysis. This analysis emphasizes and confirms a distinct immunological profile (CD8^+^ T cell sub-population, cytokines, HO-1, antibody production) for SCD patients and particularly for those undergoing CT (Fig 5).

## Discussion

Individuals with SCD are at increased risk for influenza-related complications but immune response to the influenza vaccine has rarely been investigated. As a result, some issues remain unresolved. Is post vaccination immunization comparable in SCD and healthy subjects ? Is this immunization dependent on therapy received ? Both HU and CT might induce immunomodulation [22] in those patients but little data is available about their potential impact on influenza vaccination response.

In the present study, we followed a cohort of SCD patients from the time of vaccination until six months after vaccination with the tetravalent inactivated influenza vaccine for the 2016–2017 and 2017–2018 season. Influenza A-specific antibodies, immune cells and cytokine production were explored for 72 SCD patients divided by treatment group (those receiving HU, CT, both HU and CT or none of them) and 30 healthy controls.

The influenza vaccine protects individuals from influenza virus primarily by stimulating the production of neutralizing antibodies. A response is considered protective when the HAI titer measured according to WHO guidelines is ≥ 1:40.

The majority of SCD patients included in the present study (93%) were seroprotected to the influenza A (H1N1) virus prior to vaccination compared to 60% for the control group. These findings have to be interpreted according to the history of vaccination of individuals. Indeed, all SCD patients enrolled in this study were previously immunized according to current clinical guidelines supporting influenza vaccination on an annual basis in individuals with SCD [12].

Seroprotective titers were maintained until 6 months after vaccination for 98% of SCD patients, underlining the stability in the immune response, irrespective of treatment. Achievement of seroprotection to inactivated influenza A virus vaccine for patient with SCD receiving HU has already been reported [14,23]. In contrast, in SCD patients under CT, Purohit et al. [14] documented a decrease in seroprotection after vaccination, that was not observed in our population.

Another method to assess the effectiveness of vaccination is to evaluate seroconversion defined as a four-fold rise in HAI titer. Our study demonstrates significantly lower seroconversion in SCD patients compared to healthy controls, even when patients younger than 16 years were excluded; only 22% SCD patients seroconverted following a single dose of vaccine compared to 68% healthy subjects, one month after vaccination. Impaired seroconversion was observed also at three and six months post-vaccination but no differences were observed among SCD patients’ subgroups related to treatment.

However, SCD subjects presented high residual HA-specific antibodies titer prior to vaccination and how these high levels may impact seroconversion rate remains unclear. Olafsdottir et al. [24] documented an association of high pre-vaccination titer with a negative effect on seroconversion rate. In contrast, recent data show that vaccine effectiveness is not associated with prior vaccination history [25] suggesting that the lower antibody production observed for SCD patients of the present study is independent from their previous vaccine status. It is worthy to note that we observed no significant difference in seroconversion rate among healthy subjects with high residual HA-specific antibodies or without high residual HA-specific antibodies due to a previous vaccination.

While based only on the seroconversion rate, one might thus conclude that the ability to produce anti-influenza A antibodies is reduced in SCD subjects. However, in our study, it appears that the majority of SCD patients exhibited a protective response to H1N1/2009. As it has been described by Liu et al. [26], individuals with protective titers of antibodies may still present with symptoms of influenza, therefore the seroprotection levels of SCD subjects need to be critically interpreted.

Indeed, whereas protection after influenza infection is mainly mediated by HA-specific antibodies, cell-mediated immunity also plays an active role in fighting against the virus. T-cell responses are associated with reduction of illness severity through enhancing viral clearance [27]. Early innate immune control of the influenza virus involves memory, but not naïve, CD4^+^ T cells [28]. CD8^+^ memory T cells form a first line of defence against secondary lung infections caused by influenza [29]. During a re-exposure to the viral antigens, they can rapidly acquire effector functions. Furthermore, late effector T cells (terminally differentiated), as defined by the CD8^+^CD45RA^+^CCR7^-^ profile, have been identified as a major actor of cellular immune protection against community acquired influenza illness by their direct antiviral cytotoxic potential by rapidly secreting INFγ [30].

Pediatric age-matched reference values are found in the literature for the major lymphocyte populations [19]. To avoid an eventual age bias for lymphocyte populations, analysis was performed only for individuals older than 16 years.

The decreased counts of T-cells observed in our cohort of SCD patients on CT compared to healthy donors may hamper their ability to defend against infection. We report in the present study low counts of memory CD4^+^ T cells and of memory CD8^+^ T cells (both EM and CM) for SCD patients. Given the major role of these T cell subsets in protection against influenza virus, the low counts of T cells in the SCD cohort suggests poorer response against influenza infection for SCD individuals. Counts of memory T subsets were particularly low for patients under CT as compared to other treatment subgroups. Indeed, the decrease in EM (CD8^+^), CM (CD4^+^ and CD8^+^) and TD (CD4^+^ and CD8^+^) T cells remained only significant for the subgroup of SCD patients receiving CT. These T cell defects are largely underestimated when immunization is evaluated only through anti-influenza antibodies levels.

These findings warrant further investigation, as reduced lymphocyte cell counts observed in the present work were contradictory with previously published data. Nickel et al. (7) reported elevation of most immune cell counts in patients on CT (mostly simple transfusion). In our study, CT patients were on exchange transfusion therapy and therefore results may not apply to patients on simple chronic transfusions.

We further explored the impact of the reduced T-cell subpopulations in SCD patients by a vaccine induced functional assay. Expression of the CD69 activation marker was evaluated on CD4^+^ and CD8^+^ cells following incubation with the influenza vaccine antigens to demonstrate immunological sensitization previously acquired *in vivo* by the tested individual through vaccination. T cells activation upon re-exposure to the antigen induces their proliferation and differentiation more rapidly than naïve T cells. In this study, we show a significantly poorer CD8^+^ response in SCD patients after stimulation with influenza vaccine antigens. Since CD8^+^ T cells specific to virus correlate with protection against symptomatic influenza (30), this functional *in vitro* experiment corroborates the less effective cell-immune mediated response to the virus of SCD patients compared to healthy controls.

Although higher levels of B-cells were observed in our SCD cohort, patients failed to generate sufficient B-cell memory after vaccination; 61% of SCD individuals had levels under the normal threshold value for total memory B cells. Numeration of CD27^+^ B cells is a gross estimation of B memory, though it suggests a significant defect in the ability to mount an efficient humoral immune response. This overall blunted memory B cell generation after vaccination has already been described in SCD populations [31] and may impact vaccine response, particularly for vaccines which are not frequently administrated. For these vaccines, vaccination schedule might have to be adapted.

SCD is a chronic pro-inflammatory condition characterized by elevated levels of inflammatory cytokines. We demonstrate significantly higher levels of IL-6, IL-4 and INFγ in the SCD group as reported by others [32–33]. In addition, levels of anti-inflammatory cytokines (IL-10 and TGFβ) were also higher in the SCD group compared to healthy controls. This is of particular interest as IL-10 is involved in suppressing CD8^+^ T cell response during influenza virus infection enhancing mortality and increasing virus load in the lungs [34]. We further investigated a cytokine ratio reflecting the Th1-cytokine type response, mainly involved in the anti-influenza mechanism, relative to the Treg-cytokine type activity (INFγ/IL-10). This INFγ/IL-10 ratio was significantly lower in the SCD group compared to healthy donors suggesting a cytokine imbalance after influenza vaccination. The significant decrease in Th1-cytokine type response (INFγ) and the increase in Treg-cytokine type activity (IL-10) observed in SCD patients could further negatively impact their ability to protect from influenza virus. Low levels of INFγ/IL-10 ratio have been documented in subjects with laboratory confirmed influenza among vaccinated subjects and correlated with illness severity [35].

This altered immunoregulation observed in SCD patients with an imbalance in cytokine secretion may be due to an underlying inflammatory state [36]. A recent model has been hypothesized by Yazdanbakhsh [36], where HO-1 levels/activity would impact cytokines secretion and Treg/T effector cells ratio. HO-1 is an enzyme induced by heme and upregulated in SCD patients [37]. The authors suggest a critical role of HO-1 to switch the activity from proinflammatory (high Teff/low Treg) to immunoregulatory (high Treg/low Teff) [38] which may lower the risk of allo-immunisation in SCD patients. High levels of HO-1 together with low INFγ/IL-10 ratios were observed in our SCD cohort. Patients undergoing CT presented also higher Treg levels. These trends suggest an immunoregulatory role of HO-1 also on cell-mediated vaccine response. Further studies are necessary to establish a correlation with humoral response. Moreover, the number of patients on CT in this study was relatively low and a greater inclusion of patients in the CT subgroup should be considered to corroborate this hypothesis.

## Conclusion

Protection of individuals against influenza virus involves both humoral and cellular immune response. Anti-influenza seroconversion rates were strongly reduced in the subjects with SCD. This altered vaccine response seems to be compensated by the current annual vaccination guideline as the majority of the SCD patients included in the present study mounted adequate antigen specific HAI protective titers.

SCD patients also present a discrepancy in immune cell counts as compared to healthy controls. Memory lymphocyte subsets were diminished, particularly for SCD patients receiving CT. These results, together with the cytokine imbalance documented in this work, suggest a poorer ability of these patients to fight against influenza infection.

More studies are necessary to evaluate how this impaired biological immune profile correlates with worsening clinical parameters.

Overall, our data support current clinical practice for annual influenza vaccination but we emphasize the need to investigate immunogenicity to other vaccines, particularly to those involving immunological memory.
